# Light-induced modulation of viscoelastic properties in azobenzene polymers

**DOI:** 10.1515/nanoph-2023-0728

**Published:** 2024-01-04

**Authors:** Stefano Chiodini, Fabio Borbone, Stefano L. Oscurato, Pablo D. Garcia, Antonio Ambrosio

**Affiliations:** Center for Nano Science and Technology, Fondazione Istituto Italiano di Tecnologia, Via Rubattino 81, 20134, Milan, Italy; Department of Chemical Sciences, University of Naples “Federico II”, Via Cinthia Complesso Universitario di Monte Sant’Angelo, Via Cintia, 80126 Naples, Italy; Physics Department “E. Pancini”, University of Naples “Federico II”, Via Cinthia Complesso Universitario di Monte Sant’Angelo, Via Cintia, 80126 Naples, Italy; BYM-Ingema, Centro de Empresas del Caudal, Polígono Vega de Arriba, 33600, Mieres, Spain

**Keywords:** azopolymer, nanomechanics, bimodal AFM, Young modulus, viscosity

## Abstract

Photo-induced isomerization of azobenzene molecules drives mass migrations in azopolymer samples. The resulting macroscopic directional photo-deformation of the material morphology has found many applications in literature, although the fundamental mechanisms behind this mass transfer are still under debate. Hence, it is of paramount importance to find quantitative observables that could drive the community toward a better understanding of this phenomenon. In this regard, azopolymer mechanical properties have been intensively studied, but the lack of a nanoscale technique capable of quantitative viscoelastic measurements has delayed the progress in the field. Here, we use bimodal atomic force microscopy (AFM) as a powerful technique for nanomechanical characterizations of azopolymers. With this multifrequency AFM approach, we map the azopolymer local elasticity and viscosity, with high resolution, after irradiation. We find that, while in the (previously) illuminated region, a general photo-softening is measured; locally, the Young modulus and the viscosity depend upon the inner structuring of the illuminating light spot. We then propose a possible interpretation based on a light-induced expansion plus a local alignment of the polymer chains (directional hole-burning effect), which explains the experimental observations. The possibility to access, in a reliable and quantitative way, both Young modulus and viscosity could trigger new theoretical–numerical investigations on the azopolymer mass migration dynamics since, as we show, both parameters can be considered measurable. Furthermore, our results provide a route for engineering the nanomechanical properties of azopolymers, which could find interesting applications in cell mechanobiology research.

## 1 Introduction

Azopolymers constitute the preferential azobenzene-containing material platform, where an amorphous polymer is used as an inert hosting matrix for azobenzene molecules that can be linked to the main chain through different chemical interactions [1], [2]. When an azopolymer film is exposed to two interfering coherent light beams in the UV/visible wavelength range, a photo-induced mass migration takes place because of the *trans–cis* photo-isomerization phenomenon [3], [4], [5], [6], [7]. This mass transfer translates into surface relief gratings (SRGs), which, for a p–p light polarization, can reach hundreds of nanometers of topographical modulation [8], [9], [10]. For this reason, azopolymers have been widely used to tailor chemical, optical, and mechanical sample properties [3], [11] and applications span from information storage [12] to lithography [13], [14], [15], solar energy storage [16], actuators [17], [18], photonics [19], [20], [21], [22], and photo-pharmacology [23]. Nonetheless, despite almost thirty years of investigations on azopolymer SRGs [4], [5], [7], the molecular mechanisms driving the mass migration are still under debate [3], [24], [25]. Several models have been proposed: the pressure gradient force model [26], [27], the optical gradient force model [28], the anisotropic diffusion model [29], [30], and the opto-mechanical stress model [31], [32], [33] are among the most important. However, none of them seems to be general [3], [24], since, for instance, hole-burning effects, mass-transport saturations, and spiral-shaped surface reliefs are not simultaneously described [3], [32], [34].

A possible path to unveil azopolymers mass migration mechanisms is represented by the measurement of their mechanical properties. Several studies documented in the literature have delved into the nontrivial evolution of the azopolymer elasticity under illumination. Furthermore, the viscosity of the azopolymer samples has also been experimentally examined *via* rheological macroscopic measurements [35]. The methods employed in these investigations include ultrasonic force microscopy (UFM) [36], pulse force microscopy (PFM) [37], atomic force microscopy (AFM), force-spectroscopy based techniques such as force modulation (FM) [38] or peak-force (PF) [39], and nano-indentation (NI) [40], [41]. However, all these techniques suffer from specific disadvantages such as lack of an accurate and precise viscoelastic quantification or time-consuming procedures. Likely, the lack of a nanomechanical characterization technique has possibly hindered relevant details on the mass migration phenomena. In this regard, bimodal AFM [42] provides fast, high-resolution, nondestructive, quantitative, wide-range (1 MPa–100 GPa) nanomechanical characterizations under different environments (from air to liquids) [42], [43], [44], [45]. Bimodal AFM has been used to study nanomechanical properties of a variety of samples, from polymers [44], [46], [47], [48] to proteins [49], lipid layers [50], metal–organic frameworks [49], DNA [51], virus [52], and cells [53]. It has also been shown to be capable of atomic spatial resolution [49] and high-speed performances [54]. Additionally, bimodal AFM allows going beyond a simple elastic characterization of the sample, providing also a map of the sample viscosity at the nanoscale.

In this work, we map, *via* bimodal AFM, the nanomechanical properties – Young modulus and viscosity – of an azopolymer SRG, after irradiation. We find a light-induced spatial modulation of both physical properties, which we prove not to emerge from any cross-talk with the nonflat topography. We also measure the spatial evolution of the Young modulus in different regions of the illumination spot, addressing an increasing photo-softening effect as long as the center of the spot is approached. Our nanomechanical results can be interpreted relying on the pressure gradient model [26], [27] and photo-alignment (or directional hole-burning) [3] effect.

## 2 Bimodal AM–AM theory

Multifrequency AFM [42] relies on the presence of more than one cantilever resonance (or mode). Due to the three-dimensionality of the cantilever, it can be proved that the resonance usually considered in the standard tapping mode is only the first of many resonances [55]. In the specific case of bimodal AFM, the first and second resonance are excited together, allowing to access nanomechanical material properties.

**Figure 1:**
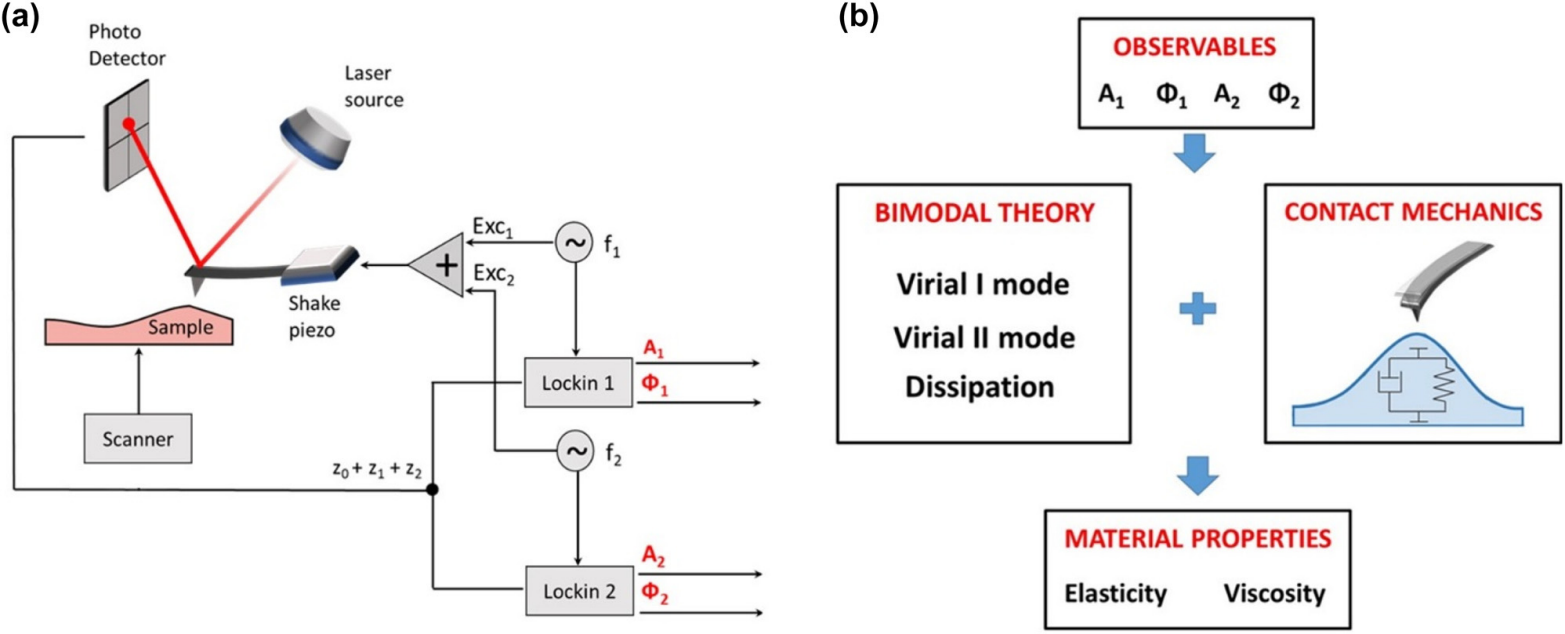
Bimodal AFM scheme and simplified theoretical framework. (a) Bimodal AM–AM set-up. The four main observables, (*A*_1_, Φ_1_, *A*_2_, Φ_2_) are highlighted in red. (b) Scheme of the transformation of the bimodal AFM observables into nanomechanical sample properties.

Several bimodal AFM set-up have been proposed depending on the feedback scheme (amplitude modulation (AM), frequency modulation (FM), or phase modulation (PM)) applied to the first and second mode [56]. In this contribution, we rely on the simplest scheme, bimodal AM–AM, to measure the viscoelastic properties of an azopolymer film. In bimodal AM–AM [57], the first mode follows an amplitude modulation scheme with the set-point kept fixed by the feedback to track the sample topography. The second mode, instead, is free to oscillate under the tip–sample force (and its gradient) providing two additional observables with respect to normal tapping mode, *i.e.*, the second mode amplitude (*A*_2_) and its phase (*Φ*_2_). We report a scheme of the bimodal AM–AM set-up in Figure 1.

We describe the motion of the cantilever system by the elastic beam equation of a rectangular cantilever [55], *i.e.*, the modified Euler–Bernoulli equation. Hence, the single modes motion follows this equation, *i* = 1, 2:

(1) $$\frac{k_{i}}{{\left(2\pi f_{i}\right)}^{2}}{\ddot{z}}_{i}+\frac{k_{i}}{2\pi f_{i}Q_{i}}{\dot{z}}_{i}+k_{i}z_{i}=F_{i}\cos\left(2\pi f_{i}t\right)+F_{ts}\left(t\right)$$

where *f*_*i*_, *Q*_*i*_, *k*_*i*_, and *F*_*i*_ are the driving frequency, the quality factor, the spring constant, and the driving force of the *i*th mode, respectively, and *F*_*ts*_ is the tip–sample force. *z*_*i*_ is the *i*th mode deflection.

The vertical time-dependent motion *z*(*t*) of the tip can be approximated by a static component *z*_0_ plus the first and second mode deflections, *i.e.*, *z*_1_ and *z*_2_:

(2) $$\begin{array}{ll} z\left(t\right) & =z_{0}+z_{1}\left(t\right)+z_{2}\left(t\right)\\ & \approx z_{0}+A_{1}\cos\left(2\pi f_{1}t-\Phi_{1}\right)+A_{2}\cos\left(2\pi f_{2}t-\Phi_{2}\right) \end{array}$$

where *A*_*i*_ and Φ_*i*_ are the oscillation amplitude and phase shift of the *i*th mode.

To correlate the nanomechanical sample properties (sample Young modulus *Y*_*s*_ and compressive viscosity *η*_*comp*_) and the bimodal AM–AM observables (*A*_1_, Φ_1_, *A*_2_, Φ_2_), we rely on the virial-dissipation energy expressions [48], [56], [58]:

(3) $$V_{1}=f_{1}\overset{1/f_{1}}{\underset{0}{\int }}F_{ts}\left(t\right)z_{1}\left(t\right)dt$$

(4) $$V_{2}=f_{2}\overset{1/f_{2}}{\underset{0}{\int }}F_{ts}\left(t\right)z_{2}\left(t\right)dt\approx \frac{\overset{2}{\underset{2}{A}}}{4\pi }\overset{1/f_{1}}{\underset{0}{\int }}\frac{dF_{ts}}{dz}\left(t\right)dt$$

(5) $$E_{1}^{\mathrm{diss}}=\overset{1/f_{1}}{\underset{0}{\int }}F_{ts}\left(t\right){\dot{z}}_{1}\left(t\right)dt$$

Remarkably, through the second expression of equation (4), the virial of the second mode can be expressed through an average along the first mode oscillation, otherwise the calculation would not be tractable [59], [60], [61]. Equations (3)–(5) can be written in function of the AFM observables once equation (1) is integrated over a first mode period. Hence, virial of the *i*th mode (*i* = 1, 2) and dissipation read:

(6) $$V_{i}=-\frac{k_{i}A_{i}A_{0i}}{2Q_{i}}\cos\Phi_{i}$$

(7) $$E_{1}^{\mathrm{diss}}=\frac{\pi k_{1}A_{1}}{Q_{1}}\left(A_{01}\sin\Phi_{1}-A_{1}\right)$$

where *A*_0*i*_ stands for the free amplitude of the *i*th mode, *i.e.*, the amplitude of oscillation when the sample is far.

In the virial and dissipation energy expressions (equations (3)–(5)), the introduction of the nanomechanical properties follows from the choice of the tip–sample force *F*_*ts*_. In this work, we model the viscoelastic tip–sample interaction with a linear model based on the standard Hertzian elastic response plus the Kelvin–Voigt (KV) model, *i.e.*, a spring (elastic contribution) and a dashpot (viscous contribution) placed in a parallel configuration:

(8) $$F_{ts}=\frac{4}{3}Y_{\text{eff}}\sqrt{R}\delta^{3/2}+2\eta_{\mathrm{comp}}\sqrt{R\delta }\dot{\delta }$$

(9) $$\frac{1}{Y_{\text{eff}}}=\frac{1-\nu_{t}^{2}}{Y_{t}}+\frac{1-\nu_{s}^{2}}{Y_{s}}$$

where *Y*_*t*_ and *Y*_*s*_ are the tip and sample Young modulus; *υ*_*t*_ and *υ*_*s*_ are the tip and sample Poisson coefficients; *Y*_eff_ is the effective Young modulus of the sample; *δ* and *R* are the indentation and the AFM tip radius; and *η*_*comp*_ is the sample compressive (or longitudinal) viscosity. Standard values for *Y*_*t*_ and *υ*_*t*_ are *Y*_*t*_ = 170 GPa and *υ*_*t*_ = 0.3 [49]. For the azopolymer Poisson coefficient, we assigned *υ*_s_ = 0.36 typical of polymethyl methacrylate (PMMA). See Materials and Methods. For polystyrene (PS), *υ*_s_ = 0.34 [48].

Compressive viscosity can be shown to be related to the more common shear viscosity *η*_*shear*_ *via* the Trouton ratio, which in the case of incompressible fluids (Poisson coefficient *υ* = 0.5) is equal to three, *i.e.*, *η*_*comp*_ = 3 ⋅ *η*_*shear*_ [62], [63]. The Trouton ratio for compressible fluid is not known, to the best of our knowledge. Since azopolymers are not perfectly incompressible, in this work we will present only compressive viscosity maps.

Inserting equation (8) in the virial and dissipation formulas (equations (3)–(5)), and assuming *A*_1_ ≫ *δ*_max_ ≫ *A*_2_ [50], we obtain three equations in three unknowns (*Y*_eff_, *δ*_max_, and *η*_*comp*_) [48], [56], [59]:

(10) $$V_{1}\approx -\sqrt{\frac{RA_{1}}{8}}E_{\mathrm{eff}}\delta_{\max}^{2}$$

(11) $$V_{2}\approx \frac{A_{2}^{2}}{4\pi }\overset{1/f_{1}}{\underset{0}{\int }}\frac{dF_{ts}}{dz}\left(t\right)dt=-A_{2}^{2}\sqrt{\frac{R}{8A_{1}}}E_{\mathrm{eff}}\delta_{\max}$$

(12) $$E_{1}^{\mathrm{diss}}=\sqrt{\frac{RA_{1}}{2}}\pi \omega_{1}\eta_{\mathrm{comp}}\delta_{\max}^{2}$$

with *ω*_1_ equal to the pulsation of the first mode (*ω*_1_ *=* *2πf*_*1*_).

This mathematical system can be easily solved providing the expressions that relate the nanomechanical sample properties to measurable AFM parameters:

(13) $$E_{\text{eff}}=-\sqrt{\frac{8}{RA_{1}}}\frac{A_{1}^{2}}{A_{2}^{4}}\frac{V_{2}^{2}}{V_{1}}$$

(14) $$\delta_{\max}=\frac{A_{2}^{2}}{A_{1}}\frac{V_{1}}{V_{2}}$$

(15) $$\eta_{\mathrm{comp}}=\frac{1}{\sqrt{2RA_{1}^{3}}}{\left(\frac{V_{2}}{V_{1}}\frac{A_{1}^{2}}{A_{2}^{2}}\right)}^{2}\frac{k_{1}}{\pi Q_{1}f_{1}}\left(A_{01}\sin\Phi_{1}-A_{1}\right)$$

The effective Young modulus and compressive viscosity maps presented in this contribution were obtained from equation (13) and (15) provided a proper calibration of (*A*_01_, *A*_02_, *k*_1_, *k*_2_, *R*, *Q*_1_, *Q*_2_, *f*_1_, *f*_2_) was performed and maps of (*A*_1_, Φ_1_, *A*_2_, Φ_2_) were obtained. We remark the *sample* Young modulus not to be equal to *effective* elasticity (*Y*_eff_). The latter, indeed, can be directly obtained from the experiment *via* equation (13), but rather represent an effective elasticity coming from the deformation of *both* the sample and the tip. In order to disentangle these two contributions, and retrieve the actual *sample* Young modulus, equation (9) should be applied. The phase maps Φ_1_ and Φ_2_ were obtained following the “Asylum Research” convention setting the free phase close to 90° [64]. No bottom-effect was considered in the nanomechanical characterization of the azopolymer sample since its thickness was above hundreds of nm [50], [65]. A simplified scheme about how to obtain the material properties starting from the bimodal AFM observables is reported in Figure 1b.

## 3 Results and discussion

Our azopolymer samples were synthetized and fabricated as detailed in the Supplementary Information (SI), Section 1. In the SI, Section 2, instead, we describe the standard two beams optical set-up used to structure the surface of the azopolymer sample.

**Figure 2:**
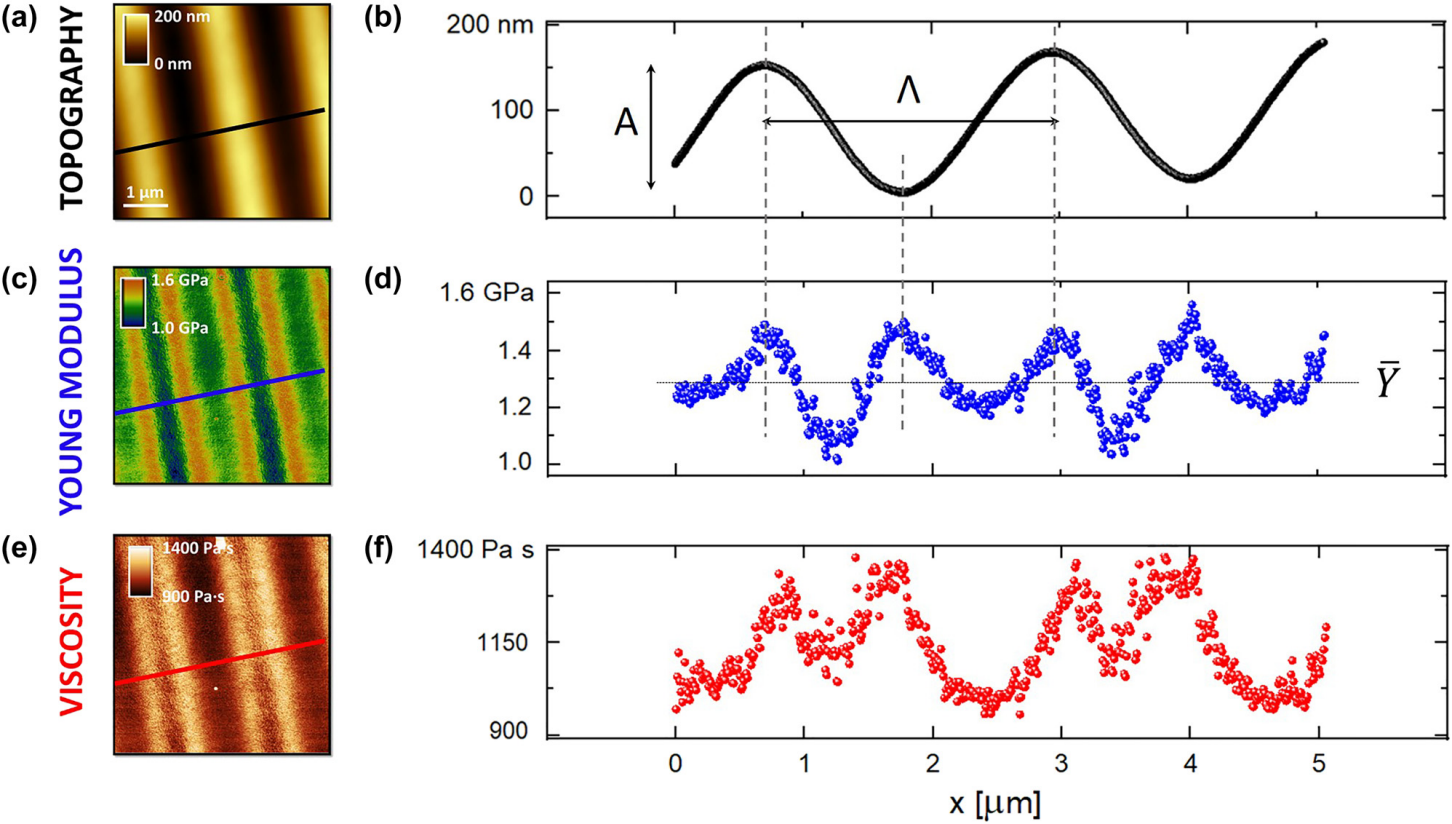
Bimodal AFM nanomechanical characterization of the azopolymer sample after irradiation. (a) 5 μm × 5 μm image reporting the topography of the sample. (b) Topography profile correspondent to the black line of Figure 2a. -A- corresponds to the peak-to-peak while Λ to the periodicity. (c) Young modulus map of the azopolymer sample in the same region of panel (a) and (d) elastic modulus profile related to the blue line of Figure 2c where average value ( $\bar{Y}$ ) and peak-to-peak (Δ*Y*) are shown. (e) Viscosity map of the azopolymer sample in the same region of panel (a) and (c). (f) Viscosity profile correspondent to the red line reported in Figure 2e. AFM parameters: *k*_1_ = 4.9 N m^−1^, *Q*_1_ = 219, *f*_1_ = 72.8 kHz, *k*_2_ = 254.2 N m^−1^, *Q*_2_ = 473, *f*_2_ = 464.8 kHz, *A*_1_ = 62.0 nm, *A*_01_ = 122.2 nm, *A*_02_ = 2.3 nm, *υ*_s_ = 0.36, *R* = 59 nm.

In Figure 2, we report the bimodal AFM measurements (more details in the SI, Section 3) that allowed collecting, at the same time, the azopolymer topography, Young modulus, and viscosity maps. It’s important to note that all these measurements were conducted *ex situ*, specifically several weeks postirradiation, and they are not intended to explore the system’s dynamics *under* illumination, a goal that would require additional experiments. Moreover, due to an azobenzene *cis*-to-*trans* transition time of about 1 day [35], [66], it is reasonable to presume that, at the time of the measurements, the majority (if not all) of the azobenzene molecules within the sample have transitioned to the *trans* state. Hence, our nanomechanical results can be considered representative of this final *trans* state, with a negligible contribution coming from the *cis* state.

We start the description of the results from the topography, Figure 2a, which shows a sinusoidal SRG with a peak-to-peak height variations *A* ≈ 150 nm, and a groove pitch Λ ≈ 2 μm (Figure 2b) matching the illumination periodicity (SI, Section 2). This experimental result is in agreement with the literature [3], [4], [5]: the irradiation of spatially structured patterns of light on azopolymer thin films produces a direct topographic modulation of the free surface of the film as consequence of a light-driven directional material transport [4], [5]. The geometry of the SRG generally depends on both the intensity [4], [5], the polarization [25], [67], and the type of wavefront [68] of the irradiated light. When intensity gradients are present in the illumination, the material motion proceeds from bright to dark zone of the pattern, with a maximum mass transfer efficiency when the polarization of the light is locally parallel to the intensity gradient. This situation occurs, for example, in p-polarized interferograms of two beams, as for the experiments in Figure 2, which are known to induce sinusoidal surface reliefs with greater amplitude than the same interference configuration with s-polarized beams [3]. We remark here that the sinusoidal profile of the topography emerges from the sinusoidal exposure dose of the interfering beams, which is here kept low enough to avoid any possible deviation from an ideal sinusoidal waveform [10], [69].

In Figure 2c, e, we provide the nanomechanical maps corresponding to the azopolymer topography in Figure 2a. The Young modulus map, Figure 2c, still shows a periodic behavior around an average value <*Y*> ≈ 1.3 GPa (lower than the pristine value measured outside the illuminated region of about 2.2 GPa, see later the discussion about the photo-softening effect), but this time characterized by twice the number of peaks present in the topography (Figure 2d). We notice that the position of both morphological crests and troughs exactly overlaps with the highest values of elastic moduli (about 1.6 GPa), while the hillsides appear to be softer (about 1.0 GPa). These experimental results are in agreement with a recent contribution, which exploited a different AFM nanomechanical technique, *i.e.*, peak force AFM, for an analogous characterization of SRGs [39]. Similar observations can be drawn for the viscosity map reported in Figure 2e and its correspondent line profile (Figure 2f). We stress that the viscosity values presented in this contribution are valid at the typical tapping frequency of the cantilever first mode *f*_1_ (here about 70 kHz) and may vary at different frequencies [48], [70]. Nonetheless, at frequencies close to *f*_1_, the viscosity measured follows a similar trend like the measured elasticity.

In the SI, Section 4, we show the AFM channels (Φ_1_, *A*_2_, Φ_2_) necessary for the generation of the two nanomechanical maps, see Figure 2c, e. In Section 5 of the SI, instead, we report a different sample region and its correspondent comparable nanomechanical characterization, therefore, supporting the reproducibility of the bimodal AFM measurements.

Before describing the implications of these experimental bimodal AFM results, their reliability should be discussed. As already reported in literature [71], nanomechanical measurements through force–distance curves can be altered by a nonflat sample geometry, determining a softening effect along the topography hillsides (the regions of maximum slope), eventually. This topographic cross-talk can be quantified through simple trigonometry, relating the measured sample Young modulus (*Y*) to the local topographical slope (tan *θ*) and the actual out-of-plane (*Y*_*OOP*_) elastic modulus of the sample (see SI, Section 6), *i.e*., [71]:

(16) $$Y=Y_{\mathrm{OOP}}\cdot {\left(\cos\theta \right)}^{5\;/2}$$

While in ref. [71], a constant slope, *θ*, is considered along the topography; in our case, the situation is more complex due to a sinusoidal topography profile of the azopolymer sample determining a variable slope, function of the local position *x*. We assume a sinusoidal topography function: $y\left(x\right)=\frac{A}{2}+\frac{A}{2}\sin\left(Kx\right)$ , with *A* equal to the topography peak-to-peak and *K* = 2π/Λ equal to the wave number (Λ being the grating periodicity), see Figure 2b. Then, the local angle *θ(x)* can be calculated *via* the derivative of the topographical function, as $\theta \left(x\right)=arctg\left(K\frac{A}{2}\cos\left(Kx\right)\right)$ . The out-of-plane (OOP) elastic modulus follows from equation (16), in each point *x* of the sample. It is worth noting that equation (16) holds true even for bimodal AFM measurements. The proof is detailed in the SI, Section 6.

**Figure 3:**
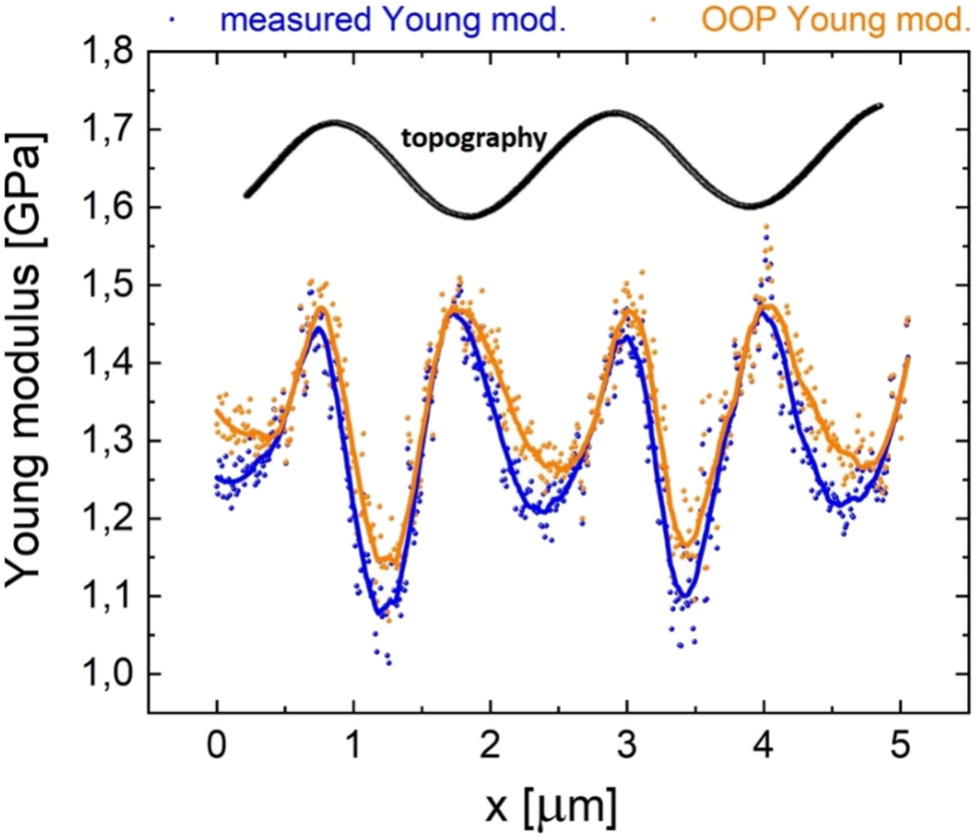
Influence of local sample slope on bimodal AFM Young modulus measurements. Measured Young modulus (blue data) and OOP elasticity (orange data) of the azopolymer sample analyzed in Figure 2. The OOP elasticity has been obtained through equation (16). A correspondent topographical line profile is also shown to localize crests, valleys, and hillsides.

In Figure 3, we report the measured Young modulus (blue data, see Figure 2c) and compare it with the correspondent OOP azopolymer elasticity (orange data) obtained from equation (16) based on the topography of Figure 2a and b. Remarkably, at the hillsides, the difference between blue and orange data is not enough to cancel the corresponding elasticity softening (a decrease of about 0.5 GPa with respect to the maximum Young modulus of crests and valleys, see Figure 2d), which, therefore, can be considered a real light-induced effect. In our specific case, in equation (16), the local angle *θ* never exceeded 10° determining a <5 % change in the Young modulus value (*A* = 80 nm, *K* = 2π/Λ, *Λ* ≈ 2 μm).

In the SI, Sections 7–11, we further support the reliability of our nanomechanical bimodal AFM measurements *via* five additional checks, specifically: (1) we compare trace and retrace profile of the main observables (Φ_1_, *A*_2_, Φ_2_) showing no difference between the two; (2) we prove the feedback loop to be fast enough to follow the spatially modulated topography; (3) we provide evidence that the tip–sample adhesion force is negligible and does not depend on which part of the SRG is considered, crest, valley, or hillside; (4) we support the application of the Kelvin–Voigt model for the measurement of the azopolymer sample viscosity by means of numerical simulations; and (5) we perform COMSOL FEM simulations to rule out any influence arising from nonlinear tip–sample interactions.

Now that we have proved the nanomechanical measurements of Figure 2 to be reliable, we provide a possible physical interpretation. According to Figure 2, we observe a higher elasticity (and viscosity) in correspondence of crests and valleys, with a relevant softening effect (30 % decrease) localized at the hillsides.

As the isomerization of azomolecules is believed to cause a local polymer expansion [26], irradiation could induce a softening of the material, whose strength is proportional to the light exposure dose. A sinusoidal light intensity profile should then cause a similar pattern in the local mechanical properties of the polymer, resulting in sinusoidal elastic modulus having the same periodicity as the illumination. This would provide a higher Young modulus in the topographic crests (dark areas) compared to the valleys (illuminated regions). Since this is not what we measure (see Figure 2c and d, where crests and valleys show the same elasticity peak), we propose the directional hole-burning effect as a second contribution to the sample stiffness, originating from azobenzene photo-reorientation under linearly polarized light [3], [39]. After many isomerization cycles, the azobenzene molecules in the *trans* state become aligned perpendicular to the direction of electric field in the illumination. As the azobenzene molecules are bonded at the sides of the polymer chains, the molecular reorientation is propagated also to the polymer [31]. Typical azopolymers are characterized by a rigid bonding angle of about 90°, finally resulting in the alignment of the polymer chain segments in the direction of light polarization. This second contribution could make the illuminated areas, *i.e.*, the topographical valleys, to correspond to regions with a high molecular orientation. This enhanced orientation would increase the van der Waals interaction between neighbored molecules providing a higher local stiffness.

The consequence of the molecular photo-alignment is the presence of a birefringence volume grating in addition to the morphological grating in SRGs. Such volume grating is responsible for the quick increase in the diffraction efficiency measured in the early stages of typical SRG inscription experiments, when the topographic grating is not formed, yet. As reported in Figure S1b, the observation of this feature in the dynamical diffraction curve recorded during the realization of the SRG used in this experiment strongly supports the presence of the volume orientation grating in our sample, partially kept inside the material matrix also when the illumination is concluded [72].

The superposition of these two contributions, *i.e.*, photo-expansion and directional hole-burning effects, qualitatively fits the experimental observation of two elasticity peaks localized at topographical crests and valleys, with the in-between hillside regions characterized by a lower Young modulus.

**Figure 4:**
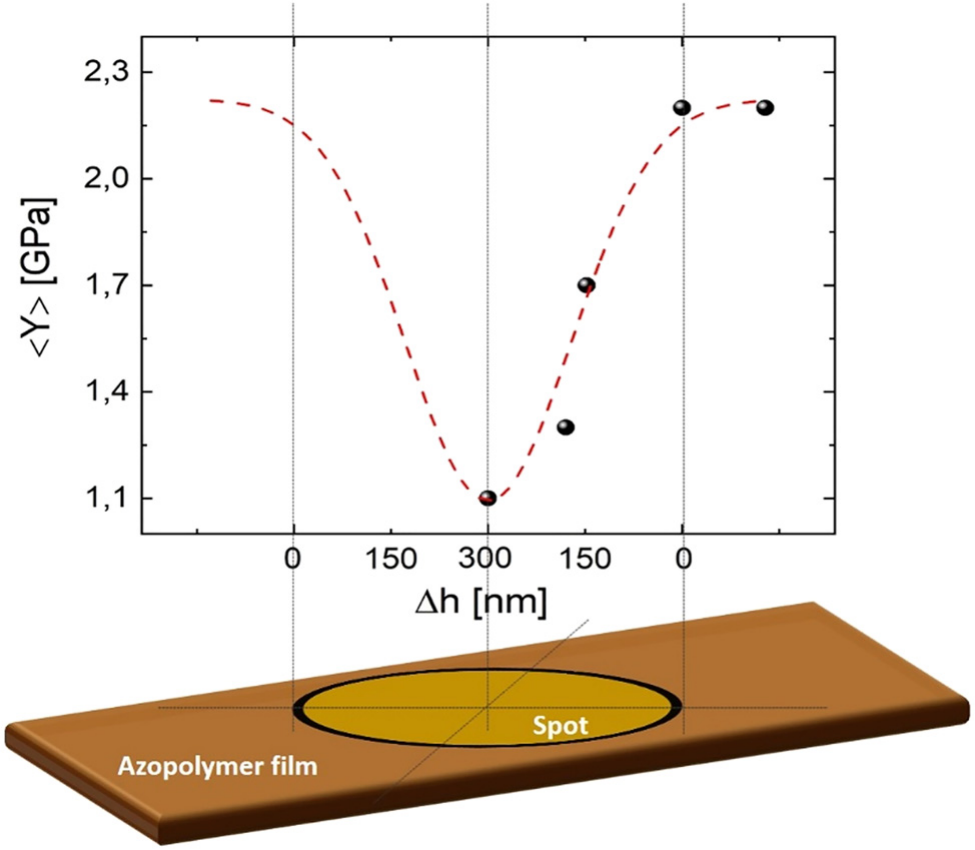
Photo-softening effect observation by means of bimodal AFM. Average Young modulus <*Y*> obtained from bimodal AFM measurements achieved in five different positions of the irradiated spot. These elasticity values are plotted in function of the topographical peak-to-peak (Δ*h*), which we have assumed to vary linearly with respect to the actual position on the irradiated spot. The error bars associated to each data (corresponding to the fitting error) are too small to be represented. The red dashed line represents a Gaussian fit of the experimental data.

The light-induced results shown in Figure 2c and d emerge from a local spatial modulation of the interference pattern restricted to an area of only 5 × 5 μm^2^. To have a more comprehensive picture of the effects of the irradiated light on the azopolymer nanomechanical response, it is worth mapping the elasticity in different regions of the irradiated spot. Qualitatively, we expect a stronger light-induced effect in the center of the spot, where the light intensity is higher, with a decreasing trend toward the edge of the spot. Nonetheless, only a quantitative nanomechanical measurement can provide a conclusive answer to the question on how the light interference pattern is actually modulating the mechanical response of an azopolymer SRG. To this end, we have used the bimodal AFM technique to measure the elasticity of the sample (after irradiation) in different regions of the irradiated spot, from the center to the final outer periphery, where pristine values should be recovered. In Figure 4, we show the results of these measurements. The *x*-axis corresponds to the topographical peak-to-peak of the SRG measured at a specific point in the illuminated spot, large in the center (about 300 nm) and small toward the edge of the spot. The *y*-axis, instead, represents the average Young modulus, <*Y*>, of the sinusoidal profile measured in that point of the spot. Indeed, following Figure 2d, we observe an approximate sinusoidal behavior of the elasticity (with half periodicity with respect to the topography) whose average can be easily obtained. This sinusoidal trend was also observed for each of the data presented in Figure 4.

Following Figure 4, the average azopolymer stiffness is spatially changing along the illuminated spot, showing a minimum at the center (about 1.1 GPa), which gradually approximates the pristine elasticity of the azopolymer film close to the spot edge (about 2.2 GPa, see later). We consider this postillumination observation as the fingerprint of the photo-softening occurring during illumination. It is worth noting that the experimental data shown in Figure 4 could be fitted by a Gaussian trend (see Figure 4, red dashed line), strongly resembling the Gaussian envelope of the interference pattern produced by Gaussian laser beams. Finally, we stress that the pristine value of the azopolymer elasticity was measured in ten different positions outside the irradiated spot, obtaining an average value (about 2.2 GPa) strongly in agreement with Young modulus of PMMA (SI, Section 12), which is expected due to the very similar chemical nature of our azopolymer [68] and, therefore, confirming bimodal AFM as a very accurate and precise tool for nanomechanical characterizations.

## 4 Conclusions

In summary, in this work we have measured the elasticity and viscosity of an azopolymer surface relief grating by means of bimodal AFM, after irradiation. The measurement is accurate and precise and provides a full viscoelastic picture of the azopolymer nanomechanical response, which could guide the community toward a better understanding of the debated mass transfer azopolymer mechanisms. For instance, following a recent contribution [73], the confirmation and quantification of the photo-softening effect here provided could be incorporated into numerical models of light-induced surface patterning. Furthermore, we foresee applications in the field of cell mechanobiology where cellular functions, such as cell migration and differentiation, are mediated by the substrate stiffness [74], [75].

## Supplementary Material

Supplementary Material Details
