# Supplementary material for: Light-induced modulation of viscoelastic properties in azobenzene polymers
Source: Nanophotonics. 2024 Jan 4;13(2):229–38. doi: 10.1515/nanoph-2023-0728 (PMC10808048; doi:10.1515/nanoph-2023-0728)
Supplement: Supplementary file 1 — Supplementary Material Details [file j_nanoph-2023-0728_suppl_001.pdf]

Supplementary Information

Light-induced modulation of visco-elastic properties in azobenzene polymers

1. Azopolymer synthesis and film preparation

Azobenzene molecules can be incorporated into a variety of material platforms, *i.e.* amorphous or liquid crystalline polymers, Langmuir-Blodgett structures(1-4) or monolayers.(5) The material used in this work is an azobenzene-containing polymer in amorphous state, bearing the azobenzene moieties as side chain of an acrylic polymeric backbone. The azopolymer was synthesized, purified and characterized as previously reported.(6, 7) For the synthesis, all the reagents were purchased from Merck and used without further purification. Proper chemical, thermal and optical characterization provided a molecular weight of Mw = 27000; a thermal phase sequence: Glass - 67 °C Nematic - 113 °C Isotropic; and maximum absorbance band at λ_{max} = 350-380 nm.

The solution for film deposition was prepared by dissolving (70 mg) the polymer in (0.50 mL) of 1,1,2,2-tetrachloroethane and filtered on 0.2 µm PTFE membrane filters. We obtained the desired film thickness (typically 1.0 ± 0.1 µm) by spin coating the solution on 24 x 60 mm cover slides at 300 rpm for 4 minutes. In the final stage, we kept the samples under vacuum at room temperature for 24h to remove solvent traces.

2. Interference set-up, photoinduced surface relief gratings (SRGs) and efficiency curve

In Fig. S1a we report the scheme of the interference setup used for SRG inscription. A horizontally (TE) polarized coherent beam at λ = 491 nm (Cobolt Calypso), is first divided into two coplanar beams of equal power by a 50:50 beam splitter (BS).

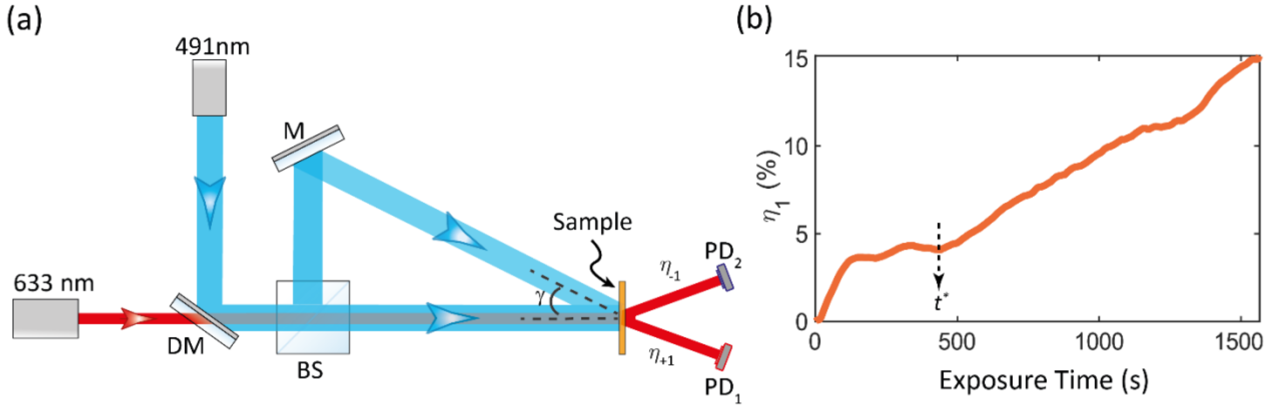

Figure S1: *(a) Schematic representation of the interference setup for the inscription of SRGs. (b) Time evolution of average first order diffraction efficiency recorded during SRG inscription (average total intensity ~ 330 mW/cm^2; exposure time 1500s).*

The two beams are then recombined on the sample surface forming an angle $\gamma \approx 14°$ that results in a grating periodicity of $\Lambda \approx 2.0$ µm, according to the relation $\Lambda = \lambda/2 \sin(\gamma/2)$. The two interfering beams had an equal power of 5.5 mW and a diameter of ~2 mm, resulting in an irradiated average intensity of the interferogram of ~330 mW/cm^2. For the AFM nanomechanical characterization, we used the grating with a total exposure time of 10 min for a topographic modulation of about 100 nm.

A TE-polarized He-Ne laser beam of 633 nm wavelength was irradiated at normal incidence into the structuring sample area to monitor in real time the diffraction generated by the developing SRG during the writing process. Two photodiodes (PD_1, PD_2) were used to record the time evolution of probe light diffracted in the +1 and -1 orders. In Fig. S1b it is shown the first order time-evolution diffraction efficiency (η_1) recorded during the SRGs inscription (average total intensity ~ 330 mW/cm^2; exposure time 1500s). The diffraction efficiency is the mean value of the +1 and -1

diffraction efficiencies recorded by the photodiodes described in panel (a). Two different regimes for the diffraction dynamics can be recognized from the curve in Fig. S1b. As reported several times, these are the consequence of the coexistence of both birefringence and topographic gratings during SRG inscription. In particular, the signal at very early stages of the photo-structuration experiments (for $t < t^*$) is determined by the birefringence grating, that quickly forms when the photo-reorientation of the azomolecules is induced by the illumination.(8, 9) At longer times ($t > t^*$), the diffraction efficiency is dominated by the morphological grating. However, in p-p illumination configurations, a significant degree of molecular and polymer chain alignment can be stored in the film even after illumination ends.(10)

3. Bimodal AM-AM

The bimodal AFM experiments were performed in air, at room temperature, using a Multimode 8 (Bruker) AFM microscope. An internal (second) lock-in was used to detect the second mode amplitude (A_2) and phase (Φ_2). PPP-FMAuD (NanoAndMore) cantilevers were used as received. The sensitivity of the first mode was obtained by performing, at the end of the experiment and without moving the laser spot focused on the cantilever, ten force-distance curves on a muscovite mica sample and calculating the average inverse slope of the contact region. Then, the spring constant of the first mode was obtained applying the standard thermal tune method.(11) The second mode of the cantilever was calibrated assuming the stiffness-frequency power law relationship, $k_2 = k_1(f_2/f_1)^{\zeta}$, where ζ is an experimental calibration parameter.(12) For PPP-FMAuD cantilevers $\zeta = 2.13$.(13) We obtained the sensitivity of the second mode through the (inverse) thermal tune method. The azopolymer AFM images were scanned at a scan rate of 0.5 Hz, with a set-point $A_1 \sim 60\,nm$ and a free amplitude $A_{01} \sim 110\,nm$. The resulting indentation was about 1 nm. Gwyddion software(14) was used to process the AFM images. The generation of the Young modulus and

compressive viscosity images was always achieved averaging trace and retrace channels of the primary maps (Φ_1, A_2, Φ_2).

The tip radius R is critical for reliable nanomechanical visco-elastic measurements. If the tip-sample force follows equation (8) indeed, both elastic and viscous contributions are R-dependent. Hence, R must be measured. In this work, we followed the already reported technique based on the measurement of the Young modulus of a reference sample.(15-17) Specifically, after the official azopolymer experiment and before the final calibration, we performed bimodal AM-AM on a polystyrene-polyolefin elastomer PS-LDPE (HarmoniX training sample, Bruker) sample focusing on the PS part. We assumed the official tip radius to be equal to the one required to obtain a PS Young modulus of 2.1 GPa.(18)

4. Additional bimodal AM-AM maps for Figure 3

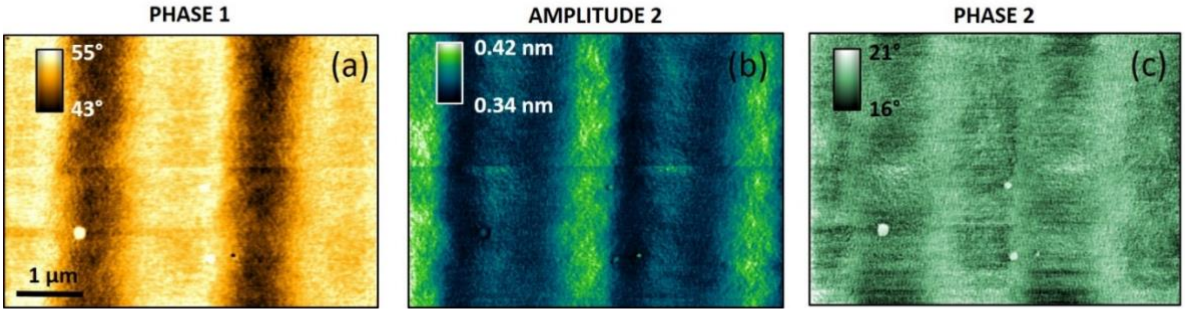

Figure S2: *(a) First mode phase map. (b) Second mode amplitude channel. (c) Second mode phase map.*

In Fig. S2 we show the three AFM channels used to reconstruct the sample Young modulus and compressive viscosity *via* Eq. (13) and (15).

5. **Bimodal AFM measurements in a different position (compared with Fig. 2) of the irradiated spot**

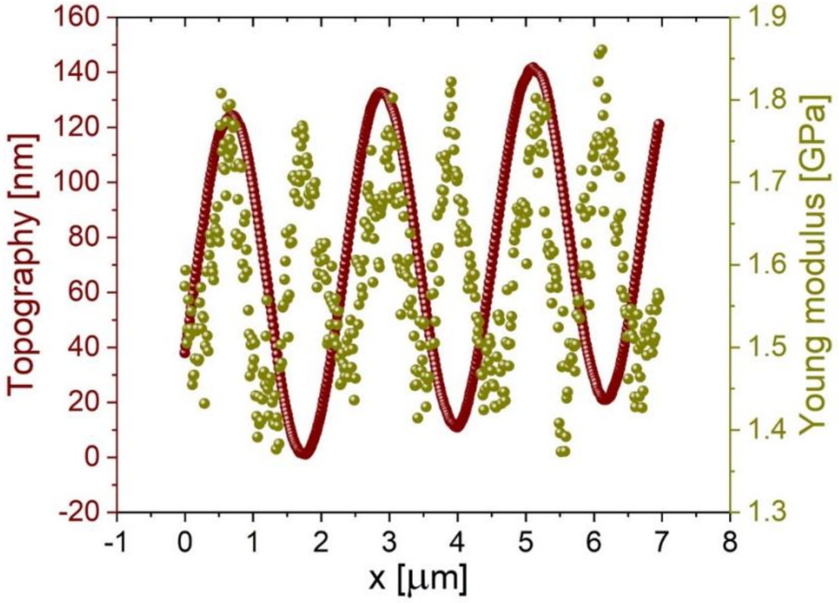

***Figure S3**: Bimodal AM-AM nanomechanical characterization of the azopolymer sample after irradiation. (a) Topography line profile (brown, left axis) showing a spatial modulation of about 120 nm. Correspondent Young modulus profile (green, right axis) of the azopolymer sample. AFM parameters: k_1 = 5.0 N·m^{-1}, Q_1 = 215, f_1 = 70.8 kHz, k_2 = 258.2 N·m^{-1}, Q_2 = 538, f_2 = 451.1 kHz, A_1 = 64.9 nm, A_{01} = 116.4 nm, A_{02} = 2.0 nm, R = 19 nm.*

6. **Sample-slope effect in bimodal AFM measurements**

Nanomechanical measurements can be affected by the sample non-flat geometry. As reported in the introduction two main AFM techniques can be considered once interested in the measurement of the sample Young modulus, *i.e.* force-distance curves and bimodal AFM. In force-distance curves, as a spectroscopic technique, the cantilever-tip system is approached and withdrawn to/from the sample in order to have, at the end of the approach part, an indentation region where the sample is strained by the AFM tip applied stress. From specific contact mechanics model it is then possible to related

the applied force to the obtained indentation and extract the sample Young modulus. The most used model follows the Hertz formula,(19) see Fig. S4a, where a flat sample is considered:

$$F_{\perp} = \frac{4}{3} Y_{eff}^{\perp} \sqrt{R} \delta_{\perp}^{3/2} \tag{S1}$$

Here, $F_{\perp}$ is the out-of-plane applied force, $Y_{eff}^{\perp}$ is the out-of-plane effective Young modulus, R is the tip radius and $\delta_{\perp}$ is the indentation obtained in the perpendicular-to-the-surface direction. Effective and real sample Young modulus are related by Equation (9) of the main text. We have written the direction perpendicular to the surface to explicit one main assumption of the Hertz model, *i.e.* the applied AFM tip force is perpendicular to the sample surface.

When a non-flat sample geometry is considered, trigonometry needs to be applied to relate the *measured* observables to their out-of-plane version. As shown in Fig. S4b, measured force F and indentation δ are related to their out-of-plane counterparts by:

$$\begin{cases} F = F_{\perp} \cos\theta \\ \delta = \frac{\delta_{\perp}}{\cos\theta} \end{cases} \tag{S2}$$

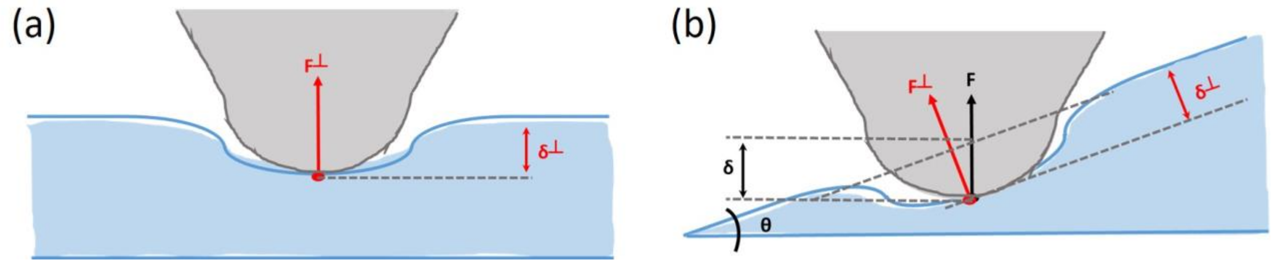

Figure S4: *(a) Representation of the indentation region once a flat sample is strained by an AFM tip. The vertical force and indentation are visualized. (b) Representation of the indentation of a tilted sample surface (constant slope = θ). The measured force and indentation are reported in black, while the out-of-plane force and indentation are shown in red.*

Inserting Eq. (S2) in Equation (S1), one obtains:

$$F = \frac{4}{3} Y_{eff} \sqrt{R} \delta^{3/2} \tag{S3}$$

with

$$Y_{eff} = Y_{eff}^{\perp}(\cos\theta)^{5/2} \quad \text{(S4)}$$

The last expression relates the measured (effective) sample elastic modulus, along the applied vertical force *F* direction, to the out-of-plane (effective) Young modulus and establishes a difference between the two *moduli*, depending on the constant slope θ.

Now, let's consider the very same problem in bimodal AFM. We are expecting Eq. (S4) to hold true even in this case, since a sample elasticity value cannot depend on the specific AFM techniques used to measure it. Following previous works,(20) it is convenient to rely on the first and second virials expressions dependent on the force F_{ts} and the indentation δ, instead of their time-dependent counterparts (equations (3) and (4)):

$$V_1 = -\frac{1}{\pi}\int_0^{\delta_{max}} \frac{dF_{ts}}{d\delta}\sqrt{2A_1}\sqrt{\delta_{max}-\delta}\,d\delta \quad \text{(S5)}$$

$$V_2 = -\frac{A_2^2}{2\pi}\int_0^{\delta_{max}} \frac{dF_{ts}}{d\delta}\frac{d\delta}{\sqrt{2A_1}\sqrt{\delta_{max}-\delta}} \quad \text{(S6)}$$

Since Equation (S2) is valid in general, we can easily insert it in equations (S5) and (S6) ending up with the following formulas:

$$\begin{cases} V_1 = V_1^{\perp}\sqrt{\cos\theta} \\ V_2 = (\cos\theta)^{3/2}V_2^{\perp} \end{cases} \quad \text{(S7)}$$

Then, from Equation (13) of the main text, we obtain:

$$Y_{eff} == -\sqrt{\frac{8}{RA_1}\frac{A_1^2}{A_2^4}\frac{V_2^2}{V_1}} = -\sqrt{\frac{8}{RA_1}\frac{A_1^2}{A_2^4}}(\cos\theta)^{5/2}\frac{V_2^{\perp,2}}{V_1^{\perp}} = Y_{eff}^{\perp}(\cos\theta)^{5/2} \quad \text{(S8)}$$

As anticipated, the expression is equal to Equation (S4).

A similar expression, this time related to a $(\cos\theta)^2$ factor, holds for the compressive viscosity (see Equation (15)).

7. Trace and retrace profiles for Fig. 3 main channels

We achieved two additional control experiments to confirm the elastic and viscous spatial modulations presented in Fig. 3 to emerge from light-structuration and not specific AFM artifacts. In Fig. S5 we report trace and retrace line profiles of the main channels involved in the Young modulus/compressive viscosity reconstructions of Fig. 2, *i.e.* ϕ_1, A_2 and ϕ_2. The full maps are reported in Fig. S2. As expected for artifact-free signals, both trace and retrace do overlap.

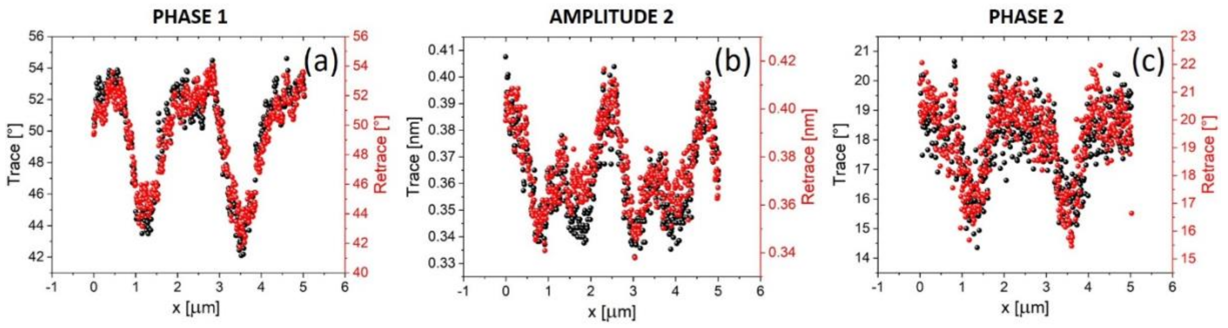

Figure S5: *(a) representative trace and retrace profiles of the first mode phase channel referred to Fig. 2. (b) Representative trace and retrace profiles of the amplitude of the second mode. (c) Representative trace and retrace profiles of the phase of the second mode.*

8. Feedback issue

In Fig. S6, we provide a proof of the proper tracking of the topography by the feedback. In panel (a) the amplitude of the first mode is reported referred to the experiment presented in Fig. 2. Since the feedback was on, the map should be as flat as possible, at a value equal to the set-point, in this case 62 nm. In panel (b) we show a representative line profile extracted from image (a): remarkably, a total oscillation of only 0.8 nm is present (out of a set-point of 62 nm). Hence, we can safely conclude the feedback not to be affected by the sample non-flat geometry.

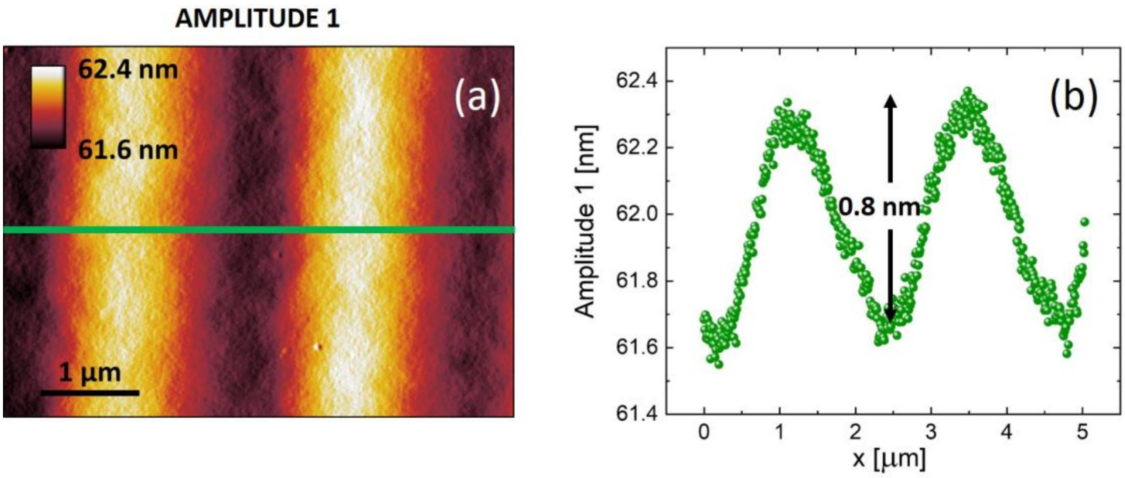

Figure S6: *(a) Amplitude of the first mode channel referred to Fig. 2. (b) Plot of the amplitude green line profile reported in panel (a).*

9. Azopolymer adhesion force

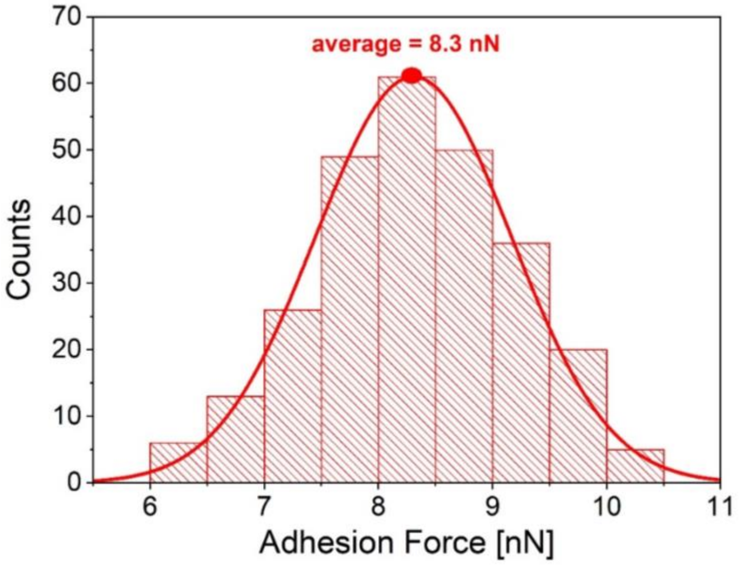

Figure S7*: Adhesion force histogram related to 300 AFM force-distance curves performed on the azopolymer sample (valley region). Crests and hillsides yielded similar values. The average adhesion force (i.e. the minima of the withdraw curve) is equal to 8.3 nN (Cantilever: PPP-FMAuD,* $k_1 = 2.7$ $N{\cdot}m^{-1}$*)*

10. Kelvin-Voigt model

To confirm the reliability of our nanomechanical results, we should do a proper discussion of the KV model. Indeed, the compressive viscosity images presented in this work, are a direct consequence of the KV model in the tip-sample force expression, see Equation (8). However, its application should not be considered general and valid for every sample, and a full knowledge on its limitations should be in hands. (20-22)

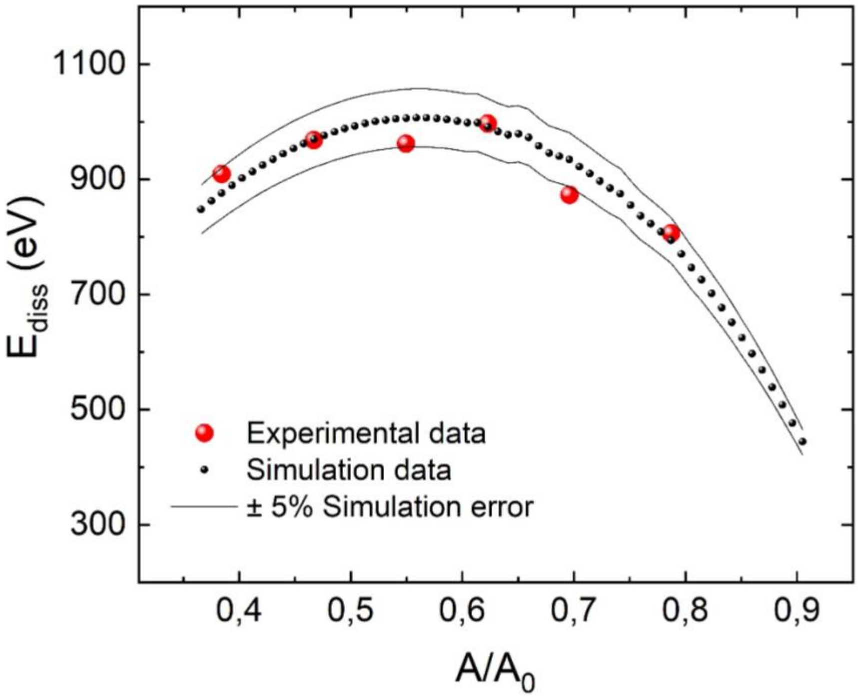

Figure S8: *Comparing of experimental dissipation (red data) and simulated (black data) values. The continuous lines address a ± 5% error in the simulated data.*

Among them we stress that: i) other dissipative phenomena could add up to the KV visco-elastic process, *i.e.* adhesion interactions,(22-24) ii) the KV model does not apply in fixed strain experiments iii) in the KV model the contact area is considered as for a Hertzian tip-sample contact.(25) and iv) generally, viscosity and elasticity are frequency dependent parameters. Hence, their values and trend presented in Fig. 2 must be considered valid only at the typical tapping frequency of the first mode.

Despite its limitations, we remark that the KV model has been involved several times in the description of the visco-elastic behavior of different polymers,(17, 20, 26-29) including PMMA.(17, 30).

Following a recent contribution,(20) we test the accuracy of the KV model by comparing experimental dissipation data with numerical simulations. Specifically, we have used the dForce program(31, 32) to simulate the cantilever dynamics when the tip experiences a Hertz + KV force model based on the measured average Young modulus and compressive viscosity (plus the experimentally calibrated AFM parameters, see the caption of Fig. 2). As reported in Fig. S8, experimental and simulated dissipation energy data agree for each experimental set-point *vs.* free amplitude ratio, *i.e.* A/A_0. Eventually, the tiny discrepancies between experimental and simulation data shown in Fig. 4, could be due to adhesion forces.(22) We have quantified (by force-spectroscopy) the average adhesion force to be about 8 nN (see SI, section 7), therefore a negligible percentage of the simulated peak force of about 100 nN.

In general, two opposite requirements should be considered in bimodal AFM: on one side the necessity to be quantitative, therefore implying relatively simple/solvable equations, and on the other side the constraint of maximum accuracy in the sample nanomechanical description. Following Fig. S8 results, we support the validity of the relatively simple Hertz + KV tip-sample force model since it is able to reproduce, reliably, dissipation energy values emerging from the sample visco-elasticity.

11. COMSOL simulations for stress-strain non-linearities

In Fig. S9 we provide a COMSOL numerical proof that the softening effect localized in correspondence of the topographical hillsides shown in Fig. 2, cannot emerge from a non-linear behavior in the stress-strain relationship typical of the azopolymer sample. Indeed, all the bimodal AFM theoretical framework presented in the manuscript, is based on a linear relationship between

stress and strain which is typically followed if the applied sample indentation is sufficiently small. To test this hypothesis, we have simulated our bimodal AFM experiments with respect to two different tip-sample geometry: a first case where the indentation is perpendicular to the sample surface (Fig. S9a) and a second case where a 10° sample slope is present with respect to the tip direction (Fig. S9b). This last case is supposed to represent the tip-sample geometry in correspondence of a hillside (see Fig. 2), while the first case is representative of topographical valleys and crests. As shown in Fig. S9a, b the FEM simulations address a similar maximum strain of 0.04 in both cases, which is below the yielding point for PMMA (0.07), *i.e.* the limit for a linear relationship between stress and strain.

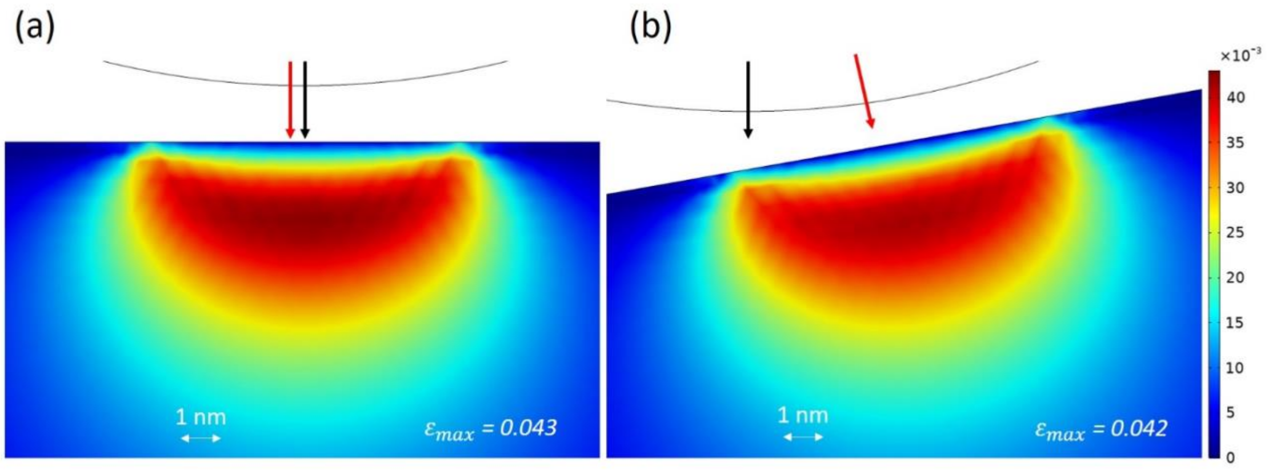

Figure S9: *FEM simulation of the maximum strain distribution for two different sample slopes. a) Flat sample: The indentation (red arrow) is parallel to the movement of the tip (black arrow). b) 10° slope sample representing a typical hillside tip-sample geometry (see Fig. 2a, b): the indentation is not parallel to the movement of the tip. In the two cases the maximum strain is similar (about 0.04) and below the yielding point (about 0.07) for PMMA. Simulation parameters: Indentation = 1nm, Tip radius = 30 nm, E = 1GPa, ν=0.36.*

12. Pristine azopolymer Young modulus

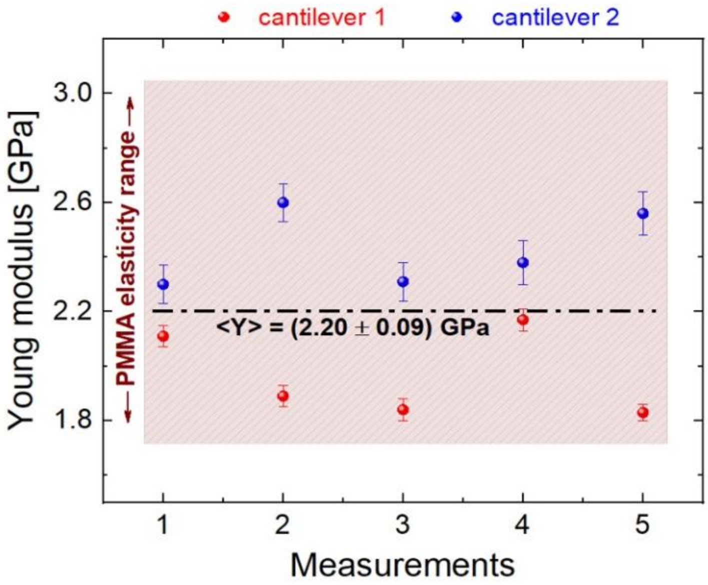

Figure S10: *Ten bimodal AFM measurements of the pristine azopolymer Young modulus value. They were achieved with two different cantilevers (1 and 2, red and blue data, respectively) having approximately the same specifications:* $k \approx 5\ N \cdot m^{-1}$, $f \approx 75$ *kHz. The average value <Y> is reported, together with the expected PMMA elasticity range.*

1. M. Haro et al., "Supramolecular Architecture in Langmuir and Langmuir−Blodgett Films Incorporating a Chiral Azobenzene," *Langmuir* **24**(18), 10196-10203 (2008).
2. M. Haro et al., "Spectroscopic Characterization and Langmuir−Blodgett Films of a Novel Azopolymer Material," *Langmuir* **23**(4), 1804-1809 (2007).
3. M. Haro et al., "Electrochemical and photoelectrochemical response of electrodes coated with LB films of an azopolymer," *Electrochim. Acta* **52**(15), 5086-5094 (2007).
4. M. Haro et al., "Photochemical behaviour of an acid-terminated azopolymer in solution and in Langmuir–Blodgett films," *Current Applied Physics* **10**(3), 874-879 (2010).
5. K. Ichimura, S.-K. Oh, and M. Nakagawa, "Light-Driven Motion of Liquids on a Photoresponsive Surface," *Science* **288**(5471), 1624-1626 (2000).
6. A. Ambrosio et al., "Light-induced spiral mass transport in azo-polymer films under vortex-beam illumination," *Nat. Commun.* **3**(1), 989 (2012).
7. S. L. Oscurato et al., "Light-Driven Wettability Tailoring of Azopolymer Surfaces with Reconfigured Three-Dimensional Posts," *ACS Appl. Mater. Interfaces* **9**(35), 30133-30142 (2017).

8. D. Y. Kim et al., "Polarized Laser Induced Holographic Surface Relief Gratings on Polymer Films," *Macromolecules* **28**(26), 8835-8839 (1995).
9. X. L. Jiang et al., "Polarization dependent recordings of surface relief gratings on azobenzene containing polymer films," *Appl. Phys. Lett.* **68**(19), 2618-2620 (1996).
10. B. Audia et al., "Influence of Photoanisotropies on Light-Controllable Structuration of Azopolymer Surface," *ACS Appl. Polym. Mater.* **2**(4), 1597-1604 (2020).
11. H. J. Butt, and M. Jaschke, "Calculation of thermal noise in atomic force microscopy," *Nanotechnology* **6**(1), 1-7 (1995).
12. A. Labuda et al., "Calibration of higher eigenmodes of cantilevers," *Rev. Sci. Instrum.* **87**(7), 073705 (2016).
13. S. Benaglia et al., "Fast and high-resolution mapping of elastic properties of biomolecules and polymers with bimodal AFM," *Nat. Protoc.* **13**(12), 2890-2907 (2018).
14. www.gwyddion.net, (July 2023).
15. S. Chiodini et al., "Bottom Effect in Atomic Force Microscopy Nanomechanics," *Small* **16**(35), 2000269 (2020).
16. X. Meng et al., "Broad modulus range nanomechanical mapping by magnetic-drive soft probes," *Nat. Commun.* **8**(1), 1944 (2017).
17. E. T. Herruzo, A. P. Perrino, and R. Garcia, "Fast nanomechanical spectroscopy of soft matter," *Nat. Commun.* **5**(1), 3126 (2014).
18. H. K. Nguyen, M. Ito, and K. Nakajima, "Elastic and viscoelastic characterization of inhomogeneous polymers by bimodal atomic force microscopy," *Jpn. J. Appl. Phys.* **55**(8S1), 08NB06 (2016).
19. H. Hertz, "Ueber die Berührung fester elastischer Körper," in *Journal für die reine und angewandte Mathematik (Crelle's Journal)*, p. 156 (1882).
20. S. Benaglia, C. A. Amo, and R. Garcia, "Fast, quantitative and high resolution mapping of viscoelastic properties with bimodal AFM," *Nanoscale* **11**(32), 15289-15297 (2019).
21. S. Benaglia, "Novel Developments and Applications of Bimodal Atomic Force Microscopy and 3D-AFM," (2021).
22. B. Rajabifar et al., "Discrimination of adhesion and viscoelasticity from nanoscale maps of polymer surfaces using bimodal atomic force microscopy," *Nanoscale* **13**(41), 17428-17441 (2021).
23. B. Rajabifar et al., "Dynamic AFM on Viscoelastic Polymer Samples with Surface Forces," *Macromolecules* **51**(23), 9649-9661 (2018).
24. E. Sahagún et al., "Energy Dissipation due to Capillary Interactions: Hydrophobicity Maps in Force Microscopy," *Phys. Rev. Lett.* **98**(17), 176106 (2007).
25. P. D. Garcia, and R. Garcia, "Determination of the Elastic Moduli of a Single Cell Cultured on a Rigid Support by Force Microscopy," *Biophys. J.* **114**(12), P2923-2932 (2018).
26. I. Chakraborty, and D. G. Yablon, "Temperature dependent loss tangent measurement of polymers with contact resonance atomic force microscopy," *Polymer* **55**(7), 1609-1612 (2014).
27. J. Waeytens, T. Doneux, and S. Napolitano, "Evaluating Mechanical Properties of Polymers at the Nanoscale Level via Atomic Force Microscopy–Infrared Spectroscopy," *ACS Appl. Polym. Mater.* **1**(1), 3-7 (2019).
28. F. Crippa et al., "Probing nano-scale viscoelastic response in air and in liquid with dynamic atomic force microscopy," *Soft Matter* **14**(19), 3998-4006 (2018).
29. A. F. Payam, A. Morelli, and P. Lemoine, "Multiparametric analytical quantification of materials at nanoscale in tapping force microscopy," *Appl. Surf. Sci.* **536**(147698 (2021).
30. Y. Ding et al., "Nanoscale mechanical characterization of PMMA by AFM nanoindentation: a theoretical study on the time-dependent viscoelastic recovery," *J. Mater. Sci.* **48**(3479-3485 (2013).
31. H. V. Guzman, P. D. Garcia, and R. Garcia, "Dynamic force microscopy simulator (dForce): A tool for planning and understanding tapping and bimodal AFM experiments," *Beilstein J. Nanotechnol.* **6**(369-379 (2015).
32. V. G. Gisbert, and R. Garcia, "Insights and guidelines to interpret forces and deformations at the nanoscale by using a tapping mode AFM simulator: dForce 2.0," *Soft Matter* **19**(31), 5857-5868 (2023).
